# Development and expert radiologist validation of a custom pipeline for simplification of oncology radiology reports using large language model

**DOI:** 10.3389/fonc.2026.1757933

**Published:** 2026-06-23

**Authors:** Prerna Garg, Neil Agarwal, Abhisht Agarwal, Vasantha Kumar Venugopal, Mahendra KM, Bharat Gupta, Jain Manish, Jitin Goyal, Radhika Daga, Sunil Kumar Puri

**Affiliations:** 1 Department of Diagnostic and Interventional Radiology, Rajiv Gandhi Cancer Institute and Research Centre, New Delhi, India; 2 Dougherty Valley High School, San Ramon, CA, United States; 3 Department of Gastro Intestinal Oncosurgery, BLK max Super Speciality Hospital, New Delhi, India

**Keywords:** colorectal cancers, custom AI pipeline, large language model (LLM), simplified reports, vernacular report summary

## Abstract

**Purpose:**

Radiology reports are often written for clinicians and contain complex medical terminology, which patients struggle to understand. This comprehension gap may lead to anxiety and misinformed decisions. This is especially important in cancer care. Large language models (LLMs) provide an opportunity to bridge this gap. Direct use of LLM by patients may potentially be misleading and may not ensure clinical fidelity. We developed an LLM- based tool to automatically translate radiology findings into clear, patient-friendly language which could improve patient-centered care.

**Objective:**

To develop and validate a bilingual LLM driven tool that simplifies oncology radiology reports into patient-understandable English and Hindi, while maintaining diagnostic fidelity and emotional tone.

**Methods:**

This study was approved by the Institute’s ethics committee. A retrospective corpus of 100 computed tomography (CT) reports (April 2025-July 2025) of patients with colo-rectal cancers were used for the development of the pipeline. Five large language models—GPT-4o, Gemini 2.5 Pro, Claude Opus, LLaMA-3.1-8B and Phi-3.5-mini—were tested. Five iterative prompt versions guided LLMs through successive refinements to ensure medical accuracy, clarity, and inclusion of a standard disclaimer. A custom pipeline; Vernacular Language Coverter(VLC), based on Gemini 2.5 Pro that takes a radiologist’s report as input and generates two patient-facing outputs: (1) a simplified English and (2) a Hindi explanation was developed. The tool thus developed was then prospectively validated using 100 de-identified reports. Simplified outputs were reviewed by two radiologists, assessing accuracy, language clarity/Terminology and readability/tone. Completeness was assessed in terms of core diagnostic completeness and minor incompleteness. Flesch Reading Ease (FRE) was calculated for a fraction of reports.

**Results:**

Mean rubric scores were high: Accuracy 4.77 ± 0.62, language clarity/Terminology 4.78 ± 0.56 and readability/tone 4.9 ± 0.32. Core diagnostic completeness was attained in all patients. 92% percent of reports were released ‘as is. Readability improved markedly (Flesch Reading Ease 49.2→73). Empathy phrases in English and Hindi were appropriate.

**Conclusions:**

An AI-driven bilingual framework significantly enhances the clarity, tone, and readability of oncology radiology reports while retaining diagnostic precision. This tool demonstrates the feasibility of safe, patient-centered communication aligned with the goals of personalized medicine.

## Introduction

Radiology reports are traditionally written using complex technical terminology that many patients find difficult to understand. With increasing adoption of digital health portals and open-access medical records, patients now routinely access their radiology reports directly, often before consultation with their treating physicians. This growing accessibility has highlighted substantial comprehension gaps, which may contribute to patient anxiety and increase the communication burden on healthcare providers (1–4).

Empathy and clarity are particularly important in oncology radiology reporting, where patients frequently experience significant emotional vulnerability. Simplified explanations may improve patient understanding and trust.

Prior studies have shown that LLMs can effectively simplify radiology reports and improve readability for patients (5–8). LLM-based systems have also shown promise in generating responses perceived as more empathetic and patient-centered than conventional physician-generated communication in certain contexts (9–11). However, important concerns remain regarding hallucinations, oversimplification, contextual inaccuracies, explainability, and broader ethical implications associated with direct clinical deployment of LLMs (11–13). Consequently, recent frameworks have emphasized the need for structured governance, clinician oversight, and adaptive human–AI collaboration in healthcare AI implementation (14, 15).

In this context, we developed a custom pipeline using LLM technology to convert oncology radiology reports into two patient-friendly formats: (1) simplified English explanations and (2) Hindi explanations. The workflow incorporated structured prompt engineering, safety-oriented guardrails, and radiologist oversight to preserve diagnostic fidelity while improving accessibility and emotional appropriateness. We specifically evaluated clinical accuracy, completeness, language clarity/terminology use and readability/tone of the generated outputs.

## Materials and methods

This single-center study used text data from de-identified retrospective and prospective Computed Tomography reports of Colorectal Cancers. Institutional ethics approval was obtained (RES/SCM/68/2025/49). We used CT reports of patients with colorectal cancers only for the development of the tool as it ensures data consistency, thus leading to improved model performance and reliability.

Model Selection: In phase I 100 Computed Tomography (CT) reports (April 2025 to July 2025) of colorectal cancer from were extracted from Hospital Information System (HIS). The prompt development and model selection process was iterative and progressive. In the initial benchmarking phase (prompt version 1, Plain-language simplification), five LLMs were evaluated on 20 anonymized radiology reports including diagnostic fidelity, Hindi fluency, API stability/latency, and cost feasibility (**Table 1**). Based on composite performance, GPT-4o and Gemini 2.5 Pro were shortlisted for further development. Iterative prompt refinement (Versions 2–5), including qualifier retention, sentence shortening, cultural tone optimization, and safety guardrails, was subsequently performed on an expanded set of 80 reports. The progressively optimized outputs were reviewed after each iteration, with Gemini 2.5 Pro consistently demonstrating superior overall performance and Hindi fluency, leading to its selection for the final pipeline. Based on the feedback of two radiologists (3- and 16-years’ experience) and one medical educator a custom software pipeline, Vernacular Language Converter (VLC) was developed which is based on Gemini 2.5 pro accessed through a secure API. This tool has a front end which is a web page which takes a radiologist’s report as input and generates two patient-facing outputs: (1) a simplified English and (2) a Hindi explanation.

**Table 1:** Initial pilot screening of LLMs across predefined evaluation domains.

Model	Reports	Diagnostic fidelity	Hindi fluency	Latency & API	Cost	Overall score	Selected
GPT-4o	20	4.20	3.80	4.50	3.20	3.93	→ V2–V5
Gemini 2.5 Pro	20	4.30	4.20	4.70	4.10	4.33	→ V2–V5
Claude Opus	20	4.10	3.60	4.30	2.80	3.70	—
LLaMA-3.1-8B	20	3.40	2.90	3.80	4.80	3.73	—
Phi-3.5-mini	20	3.10	2.60	4.20	4.90	3.70	—

**Figure 1:**
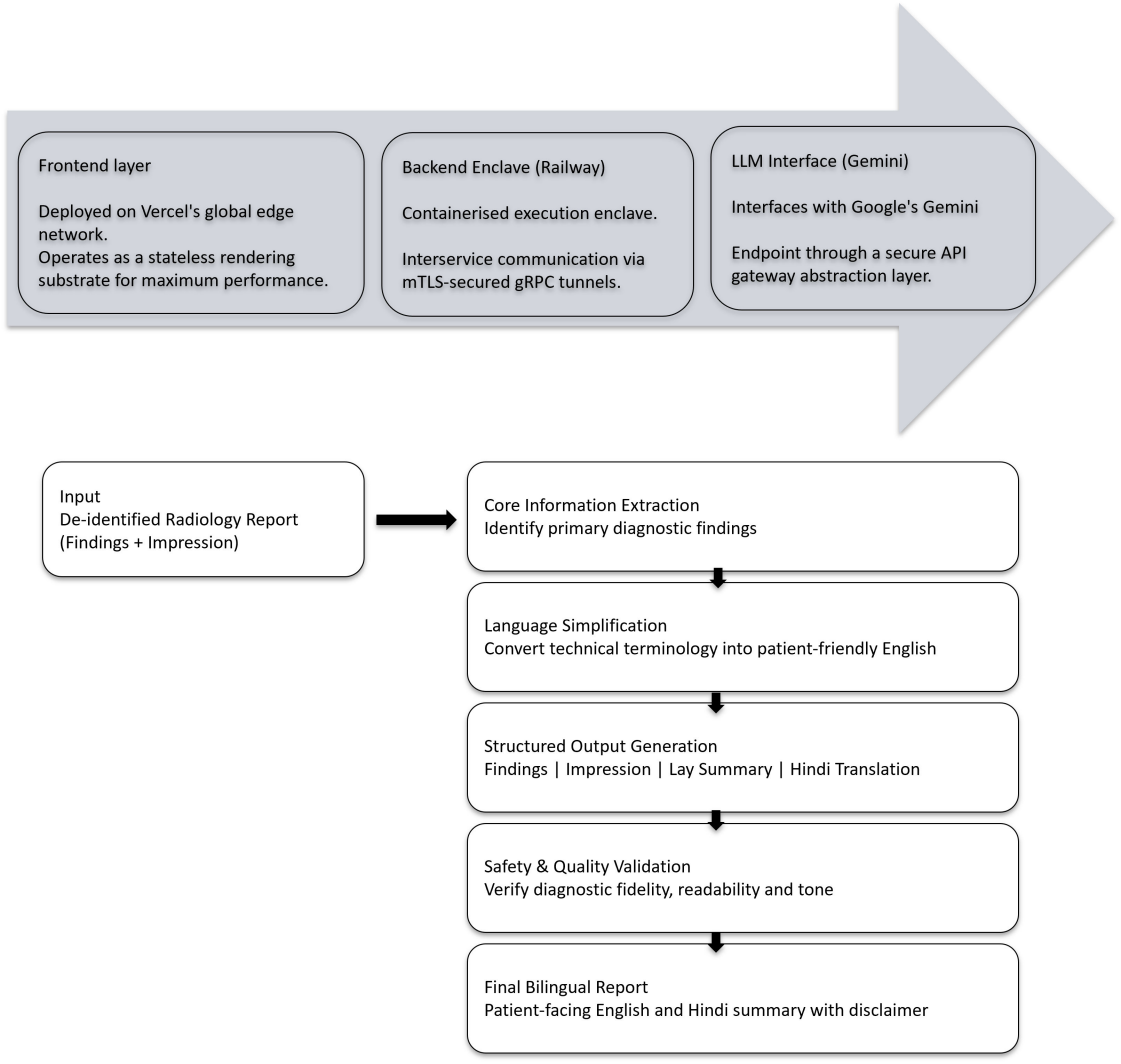
Architecture and flow chart of the custom pipeline.

**Figure 2:**
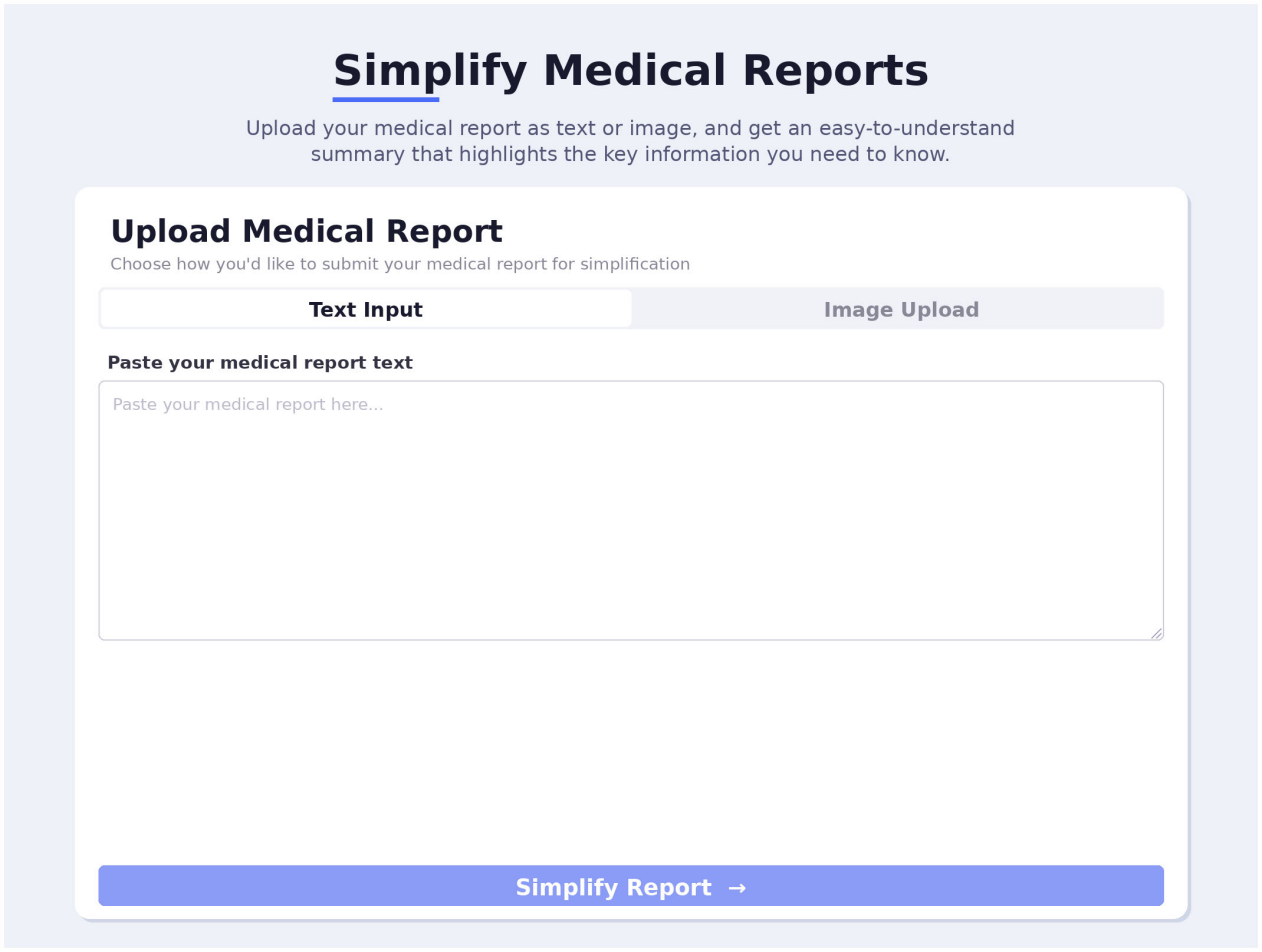
Front-end webpage of the vernacular language converter (VLC).

Architecture and security: The application’s frontend layer is deployed over Vercel’s global edge compute network, operates as a stateless rendering substrate. This layer interfaces with the Railway-hosted backend, a containerized execution enclave inter-service communication through mTLS (Mutual Transport Layer Security) secured gRPC (Remote Procedure Call) tunnels. Application interfaces with Google’s Gemini LLM endpoint through a secure API gateway abstraction layer. Data transmitted to and from the Gemini endpoint is cryptographically signed, AES-256 encrypted in transit, and compliant with zero-trust networking paradigms. The flow chart of architecture is shown in **Figure 1**. The front end is as shown in **Figure 2**.

Prompting: The VLC uses predefined prompts to guide the LLM. The first prompt instructs the LLM to act as a compassionate doctor explaining the findings in simple English (persona), avoiding medical jargon and using a reassuring tone. The prompt explicitly includes rules such as *“start with the main point, explain technical terms in everyday language, and end with advice to consult the doctor.”* Along with these instructions, the original report’s text is provided to the model. The LLM then generates a simplified English version of the report, typically in a few concise paragraphs. Next, a second prompt is applied to produce a Hindi version of the explanation. This Hindi prompt uses the same content guidelines but instructs the LLM to output in Hindi, ensuring natural phrasing and cultural appropriateness. Both prompts were written and refined through iterative experimentation. The prompts also embed a fixed medical disclaimer stating that the simplified report is for educational purposes and not medical advice.

Prompt governance and versioning: The VLC’s prompts were maintained under version control to ensure consistency and traceability. During development, we updated the prompt instructions based on radiologists’ and medical educators’ feedback (for example, adding explicit definitions for certain common terms that were confusing). All study outputs used the final prompt versions (see **Supplementary Appendices 1, 2** for representative excerpts).

Key prompt elements included: A specified persona (“medical translator explaining to a 15-year-old”), a list of do’s and don’ts (e.g. avoid jargon, start with main result, mention if tumor spread or not, advise next steps), and safety instructions (e.g. do not give treatment advice, always include a doctor consultation line). The Hindi prompt closely mirrored the English one, with added guidance on maintaining tone and clarity in Hindi. By governing the prompt content and tracking changes, we aimed to keep the VLC’s behavior consistent and aligned with clinical communication standards over time. Both the prompts as well as the tool’s parameters were adjusted to optimize performance.

Validation-In phase II 100 CT reports of Colorectal cancers were prospectively evaluated. Two senior radiologists (16- and 35-years’ experience) independently scored 100 AI-generated reports covering accuracy, completeness, language clarity/terminology and readability/tone (**Supplementary Appendix 3**).

Accuracy (fidelity) was evaluated by comparing the simplified output against the original radiology report to determine whether the generated explanation accurately preserved the intended clinical meaning without hallucinations, unsupported inclusions, omissions of major findings, or misinterpretation. Each report was scored on a 5-point Likert scale.

Completeness assessment was performed separately for Core Diagnostic Completeness and minor/incidental incompleteness. Core Diagnostic Completeness was defined as preservation of all oncologic and clinically relevant findings requiring clinical attention or management change. Omission of selected low-clinical-impact incidental findings was recorded separately as minor incompleteness. The simplification pipeline was intentionally optimized to improve patient readability and reduce information overload while preserving clinically important diagnostic information.

Language clarity and terminology use were also assessed using a 5-point scale, with emphasis on use of understandable non-technical language while preserving clinically important qualifiers. Readability and tone were similarly evaluated on a 5-point scale, including assessment of whether the generated explanations maintained an appropriate empathetic tone without causing undue alarm, anxiety, or false reassurance. Discrepancies between reviewers were resolved through consensus discussion rather than formal inter-rater reliability analysis, consistent with the exploratory and feasibility-focused nature of the study.

Additionally, Flesch Reading Ease (FRE) scores were calculated for 40% of the validation cohort to provide an objective estimate of readability improvement in the generated English summaries. As readability analysis was not the primary endpoint of the study and manual FRE calculation for all reports was resource-intensive, a representative subset was evaluated during the validation phase. The mean FRE score of the original radiology reports was compared with the mean FRE score of the corresponding AI-generated simplified English summaries to assess improvement in readability. Hindi outputs were assessed qualitatively for simplicity of language, sentence structure, vernacular appropriateness, and tone, as validated readability indices comparable to FRE are not well established for mixed vernacular Hindi medical text.

All these evaluation activities were done on reports that included a range of findings (from clear scans to complex metastatic disease) to test the VLC in varied scenarios. The evaluators were blinded to each other’s assessments to prevent bias. Any discrepancies in scoring or interpretation were subsequently resolved through consensus discussion.

Reports were classified as ‘as is’ (ready for release) or ‘needs alteration’. Evaluation outcomes were tabulated and analyzed using descriptive statistical methods including means, standard deviation frequencies, and percentages. Categorical variables (acceptable versus suboptimal output generation) were compared between English and Hindi simplified reports using Fisher’s exact test, as expected cell frequencies were less than 5. A two-tailed p-value of < 0.05 was considered statistically significant.

## Results

A total of 100 colorectal cancer computed tomography reports were assessed in validation phase including (rectum 42%, left colon,33%, right colon 25%). Clinical settings included new patients (28%), postoperative follow-up (22%), stable disease (27%), and progressive disease/recurrence (23%). The summaries generated by the tool were compared with radiologist reports.

The simplified reports were considered accurate in 97% of cases (scores 4–5) (**Table 2**). Of the three inaccurate reports, one Hindi explanation incorrectly assumed a postoperative status (hallucination), one English explanation misinterpreted postoperative thickening as disease recurrence, and one English/Hindi explanation inaccurately interpreted urinary bladder wall thickening as disease progression (incorrect inference).

**Table 2:** Table representing evaluation matrix scores in terms of mean and SD.

Parameter	Mean	SD	Interpretation
Accuracy of Findings	4.77	0.62	High accuracy
Clarity of Language and Terminology	4.78	0.56	Excellent readability
Tone and Readability	4.90	0.32	Empathetic tone

Core Diagnostic Completeness was achieved in 100% of simplified reports, with no missed oncologic or clinically actionable findings. Minor incidental findings were omitted in 39% of reports, most commonly post-operative changes (12%), renal cysts (9%), organomegaly (7%), incidental lung lesions (6%), and benign bone changes (5%) (**Table 3**). These omissions were limited to low-clinical-impact findings and reflected intentional readability optimization within the simplification pipeline.

**Table 3:** Minor incompleteness (by category, %).

Category	Percentage of minor misses (%)
Post-operative changes	12%
Renal cysts	9%
Organomegaly	7%
Incidental lung lesions	6%
Benign bone changes	5%

Language clarity and terminology use were rated appropriate in 91% of reports (scores 4–5) (**Table 2**). In 9% of cases, simplified outputs were considered suboptimal in description quality. This included the three inaccurate reports described above, as well as six additional cases with terminology or explanation-related concerns. These included inadequate description of peritoneal disease in both English and Hindi, oversimplification of bowel wall thickening in Hindi using colloquial wording such as “diwaar,” use of excessively complex Hindi terminology for the gallbladder, insufficient clarification of fistulae and postoperative bed thickening, and use of the phrase “bone freckles” for bone islands in one English output.

Overall tone and readability were excellent, with 99% of reports scoring 4–5 and a mean score of 4.90 (**Table 2**). In one report incorrectly interpreted as progressive disease, the tone was considered insufficiently empathetic and required modification in English.

Overall, 92% of simplified reports were approved “as is,” while 8% required minor language edits before release. In one case with extensive peritoneal carcinomatosis, omission of a few disease sites was considered acceptable in the context of preserving readability, and the report was approved without revision.

Readability analysis demonstrated substantial improvement in English simplified reports, with mean Flesch Reading Ease scores improving from postgraduate-level complexity (49.2) in the original reports to upper-school readability level (73) after simplification (**Table 4**). Hindi translations used concise sentence structures and simple lay terminology. Empathy-related wording was appropriate in most cases.

**Table 4:** FRE (Flesch Reading Ease) calculated from radiologist report and simplified English outputs. There was an improvement in the mean FRE score by 23.8, from post graduate difficulty level to upper school readability.

Reports	Mean FRE	Ease category
Radiologist report	49.2	Fairly difficult
Simplified English	73.0	Fairly easy

When evaluating aggregate performance, 96% (96/100) of English simplified reports and 93% (93/100) of Hindi simplified reports met all predefined quality criteria. A *post-hoc* analysis utilizing Fisher’s exact test demonstrated no statistically significant difference in the generation of acceptable outputs between the two languages (*p* = 0.537), further validating the bilingual feasibility of the pipeline.

The errata have been tabulated in detail in **Table 5**.

**Table 5:** Error mapping (total number of reports with error 9/100).

Issue category	English	Hindi
Hallucination	0	1
Incorrect inference/overinterpretation	2	1
Complex terminology/difficult wording	0	1
Oversimplification of terms	1	1
Mistranslation/verbatim conversion(in general not good)	0	2
Incomplete explanation/minor omissions	1	1
Tone/context adjustment issues	1	0

## Discussion

Medical records are now increasingly accessible to patients through electronic health portals, allowing direct access to radiology reports and other healthcare information. Despite this shift toward transparent healthcare communication, relatively few studies have explored patients’ understanding of oncology radiology reports. Wieland et al. demonstrated that patient comprehension of radiology reports is often poor and that inclusion of lay-language explanations improves understanding among oncology patients and caregivers (16). Mere availability of reports without contextual explanation may further contribute to patient anxiety, particularly in oncology settings. This challenge is especially relevant in countries such as India, where substantial linguistic and health-literacy diversity exists. By translating complex radiological findings into simplified and vernacular-language explanations, LLMs may help bridge these communication gaps and improve equitable access to understandable healthcare information.

Recent advances in LLMs have demonstrated substantial promise across healthcare applications. Singhal et al. showed that LLMs can encode clinically relevant medical knowledge and perform competitively across medical reasoning tasks (17). In radiology, these capabilities have stimulated growing interest in applications such as report simplification, patient communication, workflow assistance, and educational support (5–11, 18–20). AI-driven computational frameworks are also increasingly being explored across broader oncology and cancer-analysis domains including transcriptomic inference, spatial genomic modelling, and computational cancer biology (21–23). However, despite encouraging technological progress, important concerns remain regarding hallucinations, contextual inaccuracies, explainability, and safe deployment within real clinical environments (12–14, 20). Previous studies have also noted that oversimplification or direct translation of complex medical terminology may introduce misinformation or clinically unsafe interpretations, particularly in multilingual settings (12, 20, 24). Consequently, attention has increasingly shifted from isolated model performance toward frameworks emphasizing trustworthy human–AI collaboration, governance, and clinically integrated deployment (14, 15).

In this context, we developed a custom pipeline using LLMs to generate simplified English and Hindi explanations from oncology radiology reports, incorporating iterative prompt engineering, safety guardrails, and radiologist oversight to preserve diagnostic fidelity while improving accessibility and emotional appropriateness. The present study also represents a practical application of the TRIAD framework proposed by Li et al., which identifies Trustworthy governance, Real-world clinical value, and Integrated Adaptive Deployment as foundational requirements for meaningful human–AI collaboration in healthcare (15). Rather than evaluating LLM performance in isolation, our workflow integrated AI-generated report simplification within a clinician-supervised oncology communication pathway.

The iterative prompt refinement strategy used in the present study was informed by emerging prompt-engineering methodologies emphasizing structured instruction design, qualifier preservation, and hallucination mitigation in LLM systems (25–28). Consistent with recommendations for responsible clinical AI deployment and human-in-the-loop oversight, model outputs were continuously evaluated by radiologists and progressively refined through successive prompt iterations (29, 30).

The simplified outputs demonstrated high overall accuracy (97%), while an empathetic and appropriate tone was maintained in 99% of reports. These findings are consistent with prior studies suggesting that LLM-generated simplified radiology reports can improve patient comprehension and satisfaction without substantially compromising diagnostic fidelity (5, 6). Importantly, no major clinically actionable findings were omitted in our study, although occasional hallucinations and misinterpretations were identified. These observations reinforce that, despite encouraging performance, radiologist oversight remains critical for the safe deployment of patient-facing LLM systems.

van Driel et al. developed the RadiANT platform using GPT-4o with iterative prompt engineering to simplify radiology reports into B1-level Dutch for improved patient comprehension (6). Their study demonstrated favorable accuracy and patient satisfaction outcomes, supporting the feasibility of LLM-assisted communication tools in non-English healthcare settings. Our findings extend these observations within a multilingual oncology population in a low- and middle-income country context, where vernacular vulnerability and healthcare-literacy disparities may significantly affect patient comprehension.

A supervised radiologist-guided workflow may represent the safest and most practical implementation strategy. In this model, the radiologist first finalizes the formal report, after which the report is processed through the LLM pipeline to generate simplified English and vernacular summaries. The outputs can then undergo automated or radiologist-assisted quality checks before release alongside the original report. Such a workflow may improve patient understanding while preserving physician oversight and diagnostic safety.

Our study has certain limitations. It was a single-center study and primarily involved radiologist-based evaluation without formal inter-rater reliability assessment or direct evaluation of patient comprehension and patient-reported outcomes, which are currently being assessed prospectively. Despite these limitations, the study provides a practical framework for integrating AI-assisted multilingual communication tools into oncology radiology workflows.

## Conclusion

LLM-based bilingual radiology report simplification shows strong potential for improving patient-centered communication and accessibility. With structured prompting, safety safeguards, and radiologist oversight, high diagnostic fidelity and empathetic communication can be maintained. Our findings support the feasibility of safe, clinician-guided integration of AI-assisted communication tools into real-world radiology workflows.

## Data Availability

The raw data supporting the conclusions of this article will be made available by the authors, without undue reservation.
